# A strategy for early detection of response to chemotherapy drugs based on treatment-related changes in the metabolome

**DOI:** 10.1371/journal.pone.0213942

**Published:** 2019-04-02

**Authors:** Shahil Amin, Jodi Rattner, Mohammad Reza Keramati, Farshad Farshidfar, Mairéad G. McNamara, Jennifer J. Knox, Karen Kopciuk, Hans J. Vogel, Oliver F. Bathe

**Affiliations:** 1 Arnie Charbonneau Cancer Institute, University of Calgary, Calgary, Canada; 2 Department of Surgery, Tehran University of Medical Sciences, Tehran, Iran; 3 Department of Medical Oncology, The Christie NHS Foundation Trust and Division of Cancer Sciences, University of Manchester, Manchester, United Kingdom; 4 Department of Oncology, Princess Margaret Cancer Centre and University of Toronto, Toronto, Canada; 5 Department of Mathematics and Biostatistics, University of Calgary, Calgary, Canada; 6 Department of Biological Sciences, University of Calgary, Calgary, Canada; 7 Department of Surgery, University of Calgary, Calgary, Canada; 8 Department of Oncology, University of Calgary, Calgary, Canada; Wayne State University, UNITED STATES

## Abstract

We describe a biomarker-based approach to delivering chemotherapy that entails monitoring treatment changes in the circulating metabolome that reflect efficacy. *In-vitro*, multiple tumor cell lines were exposed to numerous chemotherapeutics. Supernatants were collected at baseline and 72 hours post treatment. MTT assays were used to quantify growth inhibition. Clinical samples were derived from a phase II clinical trial of second-line axitinib in patients with advanced hepatocellular carcinoma. Sera were collected at baseline and 2–4 weeks after treatment initiation. Response to therapy was estimated by CT scan at 8 weeks. Samples were analyzed by gas chromatography-mass spectrometry to identify metabolomic changes associated with response. In vitro, we found drug-specific and generalizable patterns of change in the extracellular metabolome accompany growth inhibition. A cell death signature was also identified. This approach was also applied to clinical samples. While the in vitro signatures were detectable in vivo, a more robust signal was identified clinically that appeared within 4 weeks of administering drug that distinguished individuals with a treatment response. These changes were extinguished as tumor growth resumed. Serial monitoring of the metabolome during chemotherapy is a means to follow treatment efficacy and emergence of resistance, informing the oncologist whether to modify treatment.

## Introduction

In recent years, there has been a rapid expansion of antineoplastic drugs. The availability of more therapeutic options represents a challenge to oncologists, who must select the best treatment for any individual. In the majority of instances, oncologists must rely on best evidence to make that selection. Using this approach, only a fraction of patients responds to any chemotherapy regimen, and this fraction diminishes with successive lines of chemotherapy due to emergence of resistance. While predictive markers may refine treatment selection, there are no predictive biomarkers for most agents. Moreover, predictive biomarkers identify individuals who do not respond, but do not guarantee benefit.

An alternative approach is to develop an improved means to assess response to systemic therapy, soon after drug has been administered. Such an adaptive approach would enable the oncologist to change therapies in a timely manner, before disease progression, and before major toxicities and high costs are incurred. Currently, treatment efficacy is monitored radiographically by computed tomography (CT) scan or magnetic resonance imaging (MRI). For solid tumors, treatment effect typically appears as a reduction in tumor size [1] or tumor attenuation [2]. Radiographically evident response may not appear until a drug has been administered for several months, and repeated radiographic studies are expensive and time-consuming. Conversely, progression also appears in a delayed fashion, often after clinical deterioration. Therefore, there is a need for a simple, inexpensive, convenient test that provides relatively immediate information on the biological activity (or lack of activity) of a treatment. Biomarker-based indicators of treatment response and emerging resistance may fulfil that need.

The metabolome represents a functional portrait of the state of health of any individual; it changes rapidly and measurably with changes in state. Drug-induced alterations in the circulating and intratumoral metabolomes that are largely a consequence of pharmacological effects have been reported [3,4,5,6,7]. Effective chemotherapy is also known to have metabolic consequences, measurable as diminished avidity in a fluorodeoxyglucose-positron emission tomography (FDG-PET) scan [2,8]. Therefore, we considered that monitoring the circulating metabolome could inform on treatment response and efficacy, as well as tumor progression.

## Results

### 2.1. Drug-specific alterations in the metabolome in vitro

Our initial approach was to identify changes in the extracellular metabolome that accompanied growth inhibition *in vitro*, using gas chromatography-mass spectrometry (GC-MS). The extracellular compartment was our focus because this was thought to most closely reflect what would change in the circulation, which was pertinent to a blood test that could be used to monitor response. Cell lines were exposed to a range of doses of various antineoplastic agents with diverse mechanisms of action. After 72 hours, degree of growth inhibition was estimated by MTT assay. A “response” to any agent was considered to be present in conditions where there was ≥15% growth inhibition. By comparing changes in the metabolome that accompany growth inhibition to changes that appear in the absence of growth inhibition, patterns of changes attributable to growth inhibition can be identified.

**Fig 1:**
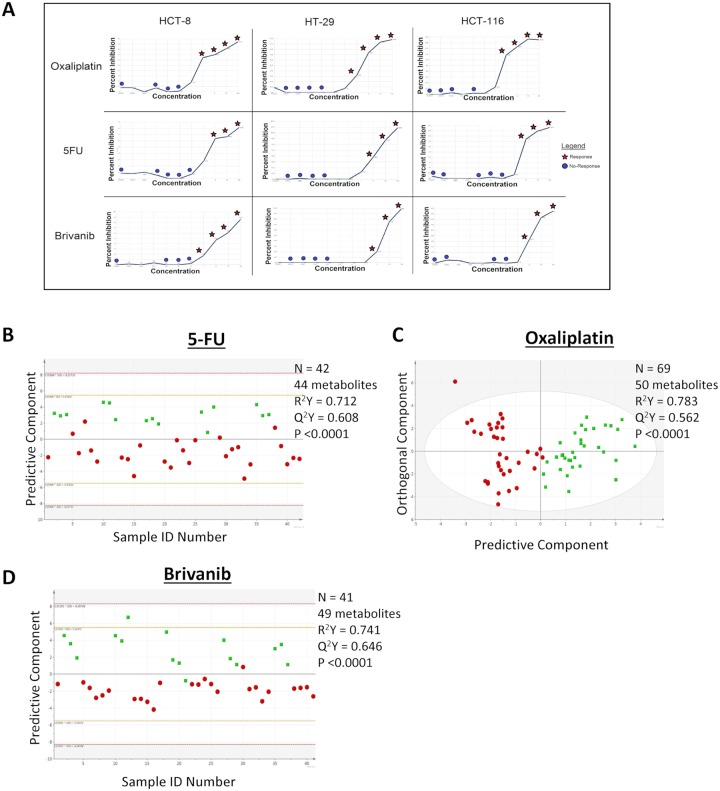
A. Dose response curves for HCT-8, HT-29 and HCT-116 Colorectal Cancer cells treated with oxaliplatin, 5-FU and brivanib. Red stars denote samples that are classified as response, and samples below our threshold value for response are denoted with blue circles. B, C, D. OPLS-DA score scatter plots illustrating differences in the metabolomic signatures associated with response (green boxes) and absence of response (red circles). Colorectal cancer cell lines were treated with 5-FU (B), oxaliplatin (C) or brivanib (D).

Three colorectal cancer cell lines (HCT-8, HT-29, and HCT116) were treated with three drugs, each with diverse mechanisms of action (5-fluorouracil (5-FU), oxaliplatin, brivanib) (Fig 1A). To identify changes in metabolites that discriminated conditions with and without growth inhibition, orthogonal partial least squares-discriminate analysis (OPLS-DA) was performed, using *changes* in metabolite concentrations as the dependent variable. For each drug, it was possible to identify a pattern of changes in the metabolome that clearly discriminate conditions in which there was or was not growth inhibition (Fig 1B to 1D). Drug-specific alterations in metabolites that specifically appear with growth inhibition are listed in Table A-C in S1 File. As expected, the lists of metabolites that change in association with growth inhibition differs with the type of drug administered. In all, these data demonstrate response-specific patterns of changes in metabolites that differ between drugs.

### 2.2. Common metabolomic changes associated with drug-induced growth inhibition

There were 8 metabolites that changed with growth inhibition from all three drugs. Therefore, it was conceivable that there existed a pattern of changes that is independent of the type of drug. First, metabolomic changes secondary to growth inhibition were identified from each of seven cell lines exposed to 2–4 different drugs. Metabolites with a variable of importance (VIP) score < 1 were removed from further analysis. There were 22 metabolites that appeared in lists from >3/7 cell lines tested (Table D in S1 File).

**Fig 2:**
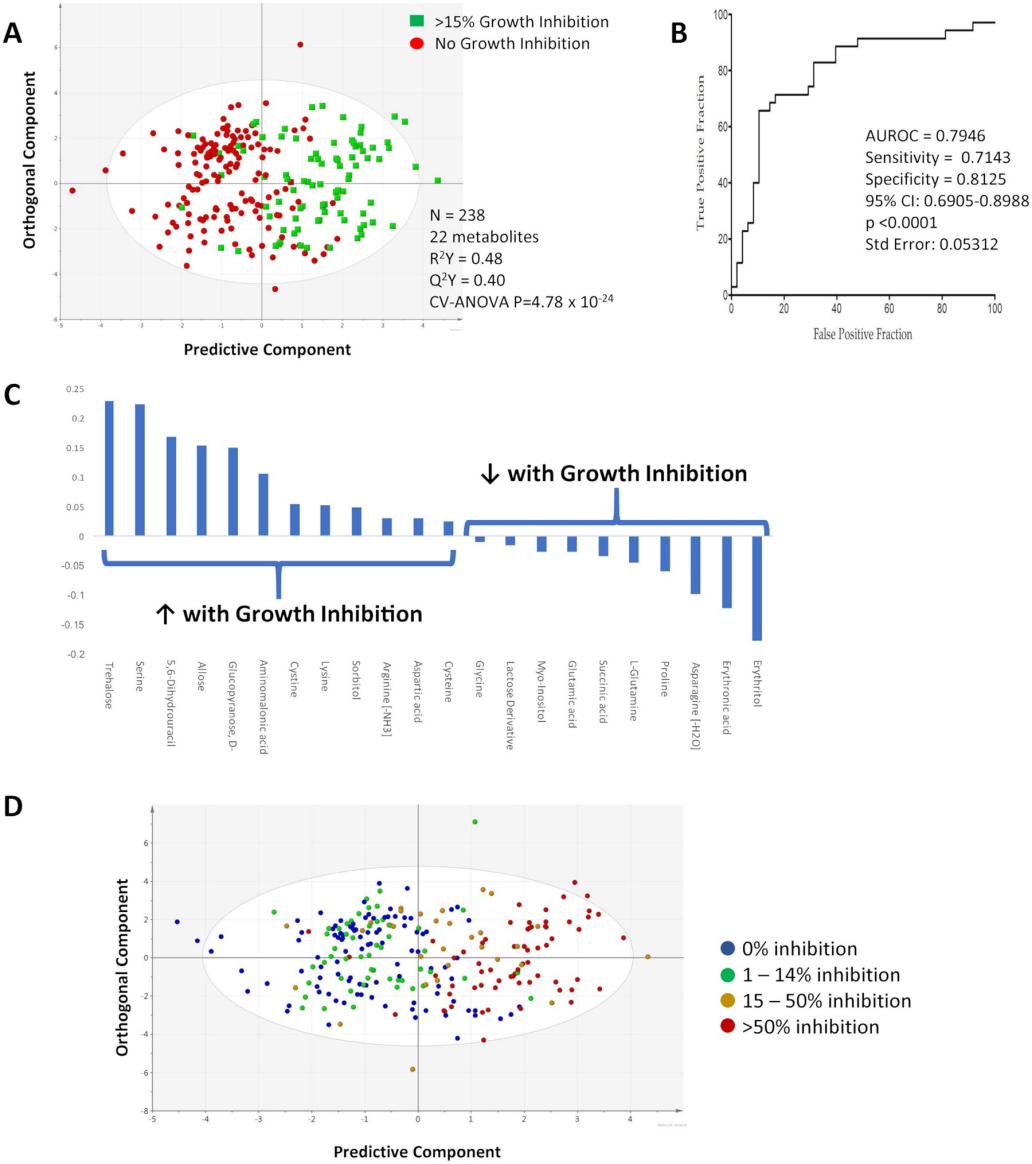
A. OPLS-DA scores plot depicting the discrimination of metabolomic profiles of supernatants derived from diverse cell lines treated with chemotherapeutic agents with a variety of mechanisms of action. B. ROC curve illustrating the performance of the 23 metabolite model in discriminating response from no-response in a separate group of 83 samples in which a variety of cell lines was treated with diverse drugs. C. Coefficient plot for metabolites that are differentially abundant in the training set from all of the tested cell lines. D. OPLS-DA supervised scores plot of response versus no-response of 238 randomly chosen samples from 7 cell lines treated with various antineoplastic agents. Samples are coloured based on percentage of inhibition.

These 22 metabolites were further applied to a training set of 238 random samples consisting of various cell lines treated with different antioneoplastic drugs with diverse mechanisms of action, at lethal and non-lethal doses (about 75% of all samples). This enabled construction of a statistically significant model that discriminated response category (Fig 2A). In this model, trehalose, serine and 5,6-dihydrouracil were the three metabolites that were most increased with growth inhibition, and erythritol, erythronic acid and asparagine were the three most increased in conditions where there was no growth inhibition (Fig 2C).

The remaining 83 samples were used as a validation set, and a receiver operating characteristic (ROC) curve was generated (Fig 2B). Where the biomarker identifies conditions in which a drug caused growth inhibition, the positive predictive value was 0.74, and the negative predictive value was 0.80. To determine if this growth inhibition signature is quantifiable (as opposed to simply present/absent), samples were stratified based on degree of inhibition. Fig 2D demonstrates that the biomarker signal is a continuum, reflecting the degree of growth inhibition.

### 2.3. Metabolomic alterations that accompany cell death

**Fig 3:**
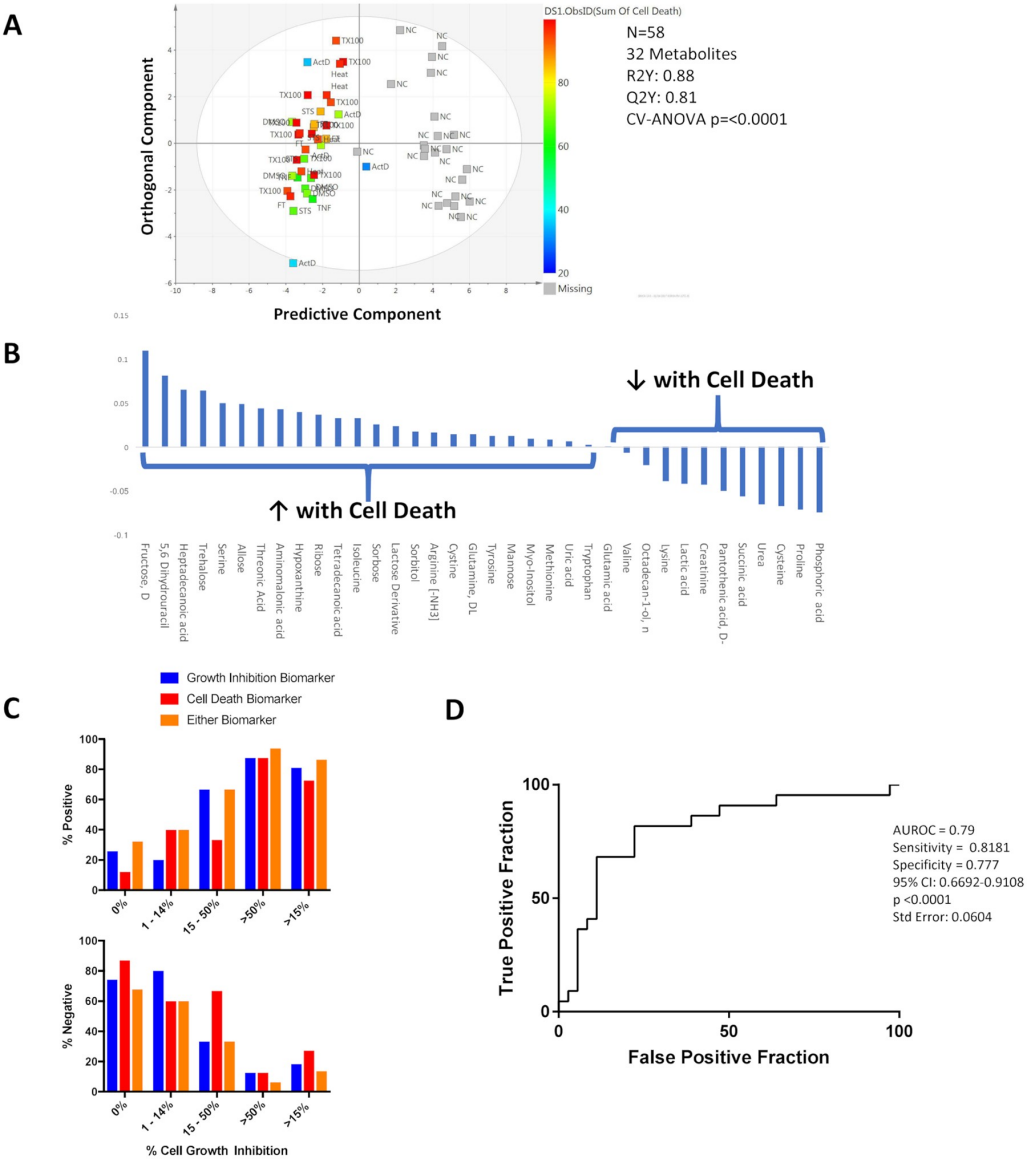
A. OPLS-DA scores scatter plot of death versus no-death in both HCT-116 and MDA-MB-231 cells. Samples are coloured based on the percentage of cell death (sum of percent apoptosis and percent necrosis). STS denotes staurosporine samples, TNF is TNF-α, ActD are actinomycin-D samples, TX100 are triton X-100 samples, FT is freeze thaw samples and NC are negative control samples. B. Coefficient plot, 32 metabolites, cell death, only named metabolites. C. Combined use of growth inhibition and cell death biomarkers in cell lines treated with various antineoplastic agents. On the left, proportion of conditions in which either of the biomarkers detects a response, as a function of the degree of cell growth inhibition. On the right, the proportion of conditions in which one or both biomarkers identifies the absence of a response. D. ROC analysis of a combined biomarker incorporating the cell death signature and response signature tested on cells treated with chemotherapeutic agents.

In the experiments described above, growth inhibition (“response”) can occur as a result of cell death or diminished proliferation. Cell death can occur by a number of different mechanisms, crudely classified as necrosis or apoptosis. Experiments were designed to identify the metabolomic changes that occur as a result of cell death, using HCT-116 colorectal cancer cells and MDA-MB-231 breast cancer cells treated with a variety of chemical and physical agents known to induce apoptosis and/or necrosis. Conditions were selected based on the presence of measurable cell death without complete destruction of cells. There were 36 metabolites that were detected in this experiment that were not detected in the drug response experiments. When all of the conditions were analyzed together, the metabolomic changes from baseline in the supernatant of cells that underwent apoptosis or necrosis were clearly distinguishable from negative controls (Fig 3A). The experimental design did not allow a reliable means to distinguish changes attributable to apoptosis with changes secondary to necrosis.

The metabolites comprising the model are summarized in Fig 3B **and Table E in** S1 File. Of the 32 metabolites that contributed to this model, fructose, heptadecanoic acid and serine were the top three metabolites increased with cell death; and phosphoric acid, proline and cysteine decreased in association with cell death (compared to negative controls). Interestingly, some of the metabolites that changed in association with cell death were also constituents of the 22-metabolite model that signified growth inhibition in cells treated with various antineoplastic drugs. Specifically, serine, aminomalonic acid, cystine, arginine, proline, succinic acid, trehalose, 5,6-dihydrouracil and glutamic acid changed from baseline in the same way. This may be related to the fact that cell death contributes to growth inhibition in some conditions.

### 2.4. The metabolomic signature of cell death is detectable in cancer cells treated with antineoplastic agents

The 32-metabolite cell death signature was applied to cells treated with various chemotherapeutic agents. To do this, the cell death model was modified, because only 29 of the metabolites in that model were also detectable in the drug response experiments. (This modified model had a R^2^Y score of 0.46, a Q^2^Y score of 0.34, and p<0.0001.) In conditions in which there was growth inhibition ≥15%, the cell death signature appeared in 53% (15/28) of samples; and in conditions with <15% growth inhibition, 83% (42/52) had no cell death signature present (Fig 3C). The AUROC that reflects the capability of the cell death signature to identify response is 0.80. The positive predictive value was 0.6, and the negative predictive value was 0.76.

Some chemotherapeutic agents are cytotoxic, and a response to such agents would hypothetically be accompanied by metabolomic changes reflected by either the response biomarker or the cell death biomarker. On the other hand, some agents (such as molecularly targeted agents) are only cytostatic, and the cell death signature may not be as apparent. We considered that applying both biomarkers to the validation group of conditions would enhance the capability to identify responders and (more importantly) non-responders. Fig 3D illustrates how well the biomarkers on their own and together identify a response or absence of response. In conditions where there was a response, at least one of the biomarkers was positive in 86.3% of samples. AUROC for the combined use of biomarkers was 0.79, negative predictive value was 0.88. The positive and negative predictive values for the combined use of biomarkers was 0.692 and 0.875 respectively, with an AUROC of 0.79. Given these findings, it is possible that reliable detection of response may involve testing for the metabolomic changes that accompany a number of processes (e.g. growth inhibition, cell death, loss of tumor signal), especially in patients treated with multiple drugs.

### 2.5. Clinical samples taken serially from patients with hepatocellular carcinoma (HCC) receiving axitinib

**Fig 4:**
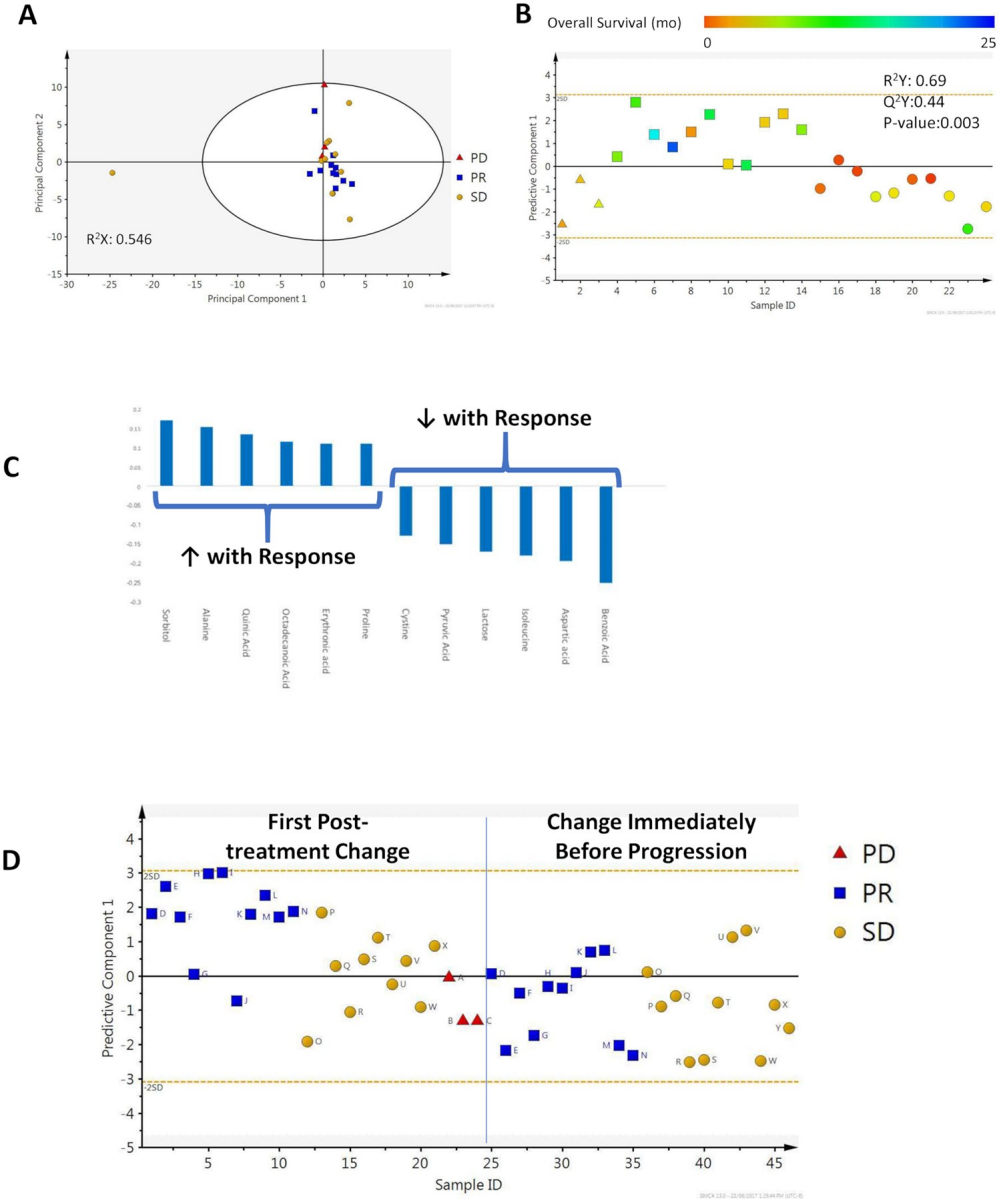
Clinical samples from patients with advanced hepatocellular carcinoma treated with axitinib. A. PCA, with response groups indicated. B. OPLS-DA scores scatter plot demonstrating the appearance of the axitinib-specific biomarker in each response category. Individuals are colored based on overall survival. C. Coefficient column plot illustrating the directionality of change of metabolites comprising the axitinib response biomarker. D. Appearance and disappearance of the axitinib biomarker for each individual from each response category: after the first treatment, and at progression.

Fasting samples taken before and during treatment with a tyrosine kinase inhibitor, axitinib, in patients with advanced HCC were tested by GC-MS [9]. Response was determined using Choi criteria^2^, since this tyrosine kinase inhibitor does not frequently cause tumor shrinkage. In this cohort, there were 3 patients with progressive disease (PD), 11 patients with a partial response (PR), and 11 patients with stable disease (SD). In general, there were more metabolites detectable in the cell line experiments than in clinical samples: 136 compounds were detectable in clinical samples, 68 were identifiable metabolites. There were no intrinsic differences between response groups, but one individual with stable disease was a clear outlier, and was excluded from further analysis (Fig 4A).

The OPLS-DA models for response and for cell death were applied to clinical samples at 2–4 weeks after initiation of axitinib. Of the 22 metabolites comprising the *in vitro* response signature, 17 were detectable in blood. When the growth inhibition and cell death biomarkers were applied to the clinical trial samples, at least one of the biomarkers was positive in 8 of 11 individuals who had a PR; in 3 of 10 patients who had SD; and none of the three patients with PD (Fig 4B). Both biomarkers were negative in 2 of 11 patients with PR, 8 of 10 patients with SD and all patients with PD. Therefore, the biomarkers were capable of categorizing patients by response group to some degree. However, when the growth inhibition model and the cell death model were directly applied to the clinical trial cohort, the relationship was not significant.

**Fig 5:**
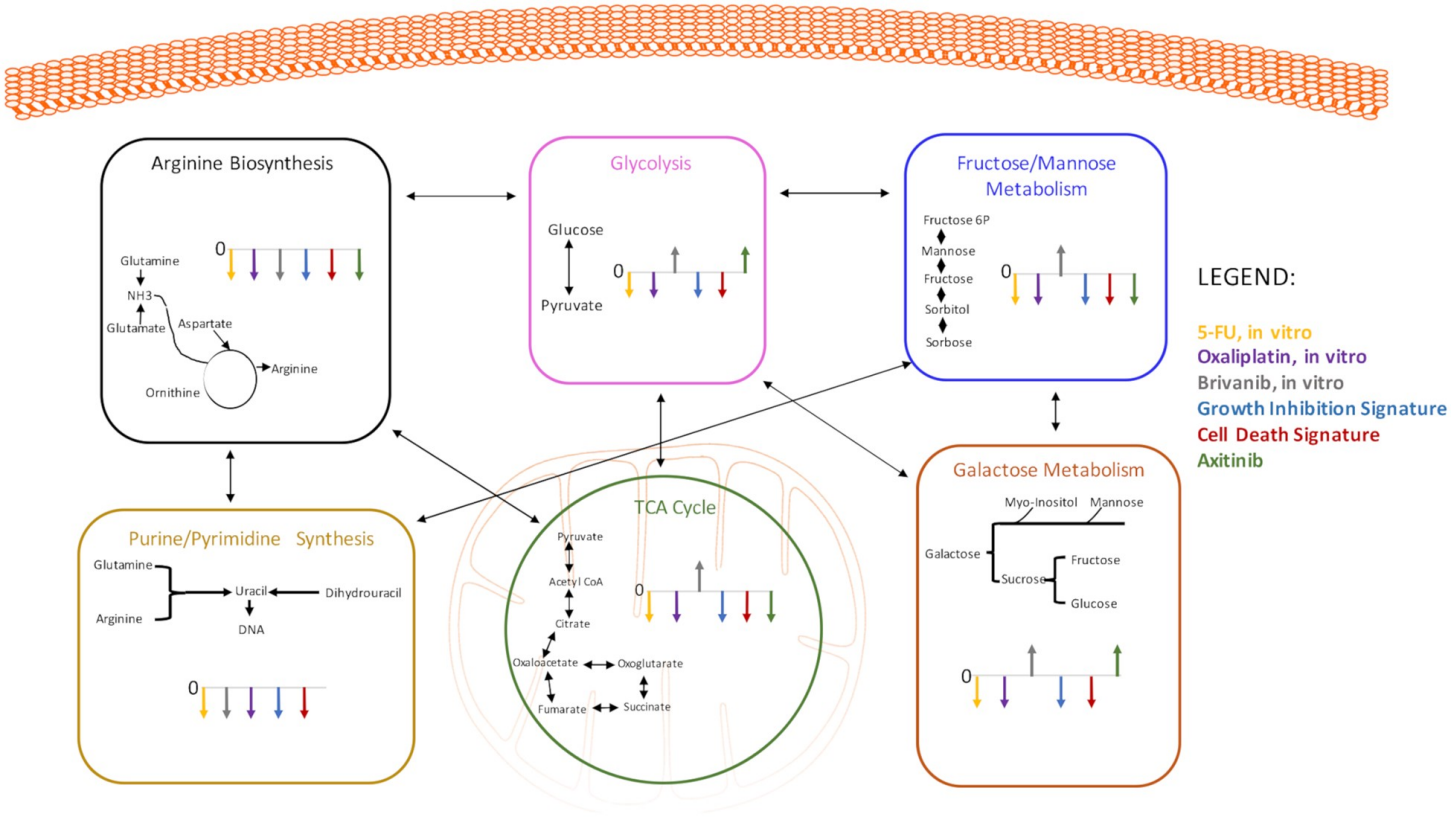
Summary of changes in metabolic pathways observed with growth inhibition; cell death; effective doses of 5-FU, oxaliplatin and brivanib; and measurable response following axitinib.

The fact that the *in vitro* metabolomic models of drug response and cell death could not be well recapitulated *in vivo* could be related to a number of factors. The *in vitro* model was based on changes at 24 hours after drug exposure, and clinical samples were taken later. The circulating metabolome is a net product of contributions from tumor, host, and environmental factors. Many of the patients in the axitinib trial had some element of liver dysfunction. Moreover, drug-specific and tumor-specific changes in the circulating metabolome may have predominated. Therefore, to explore the possibility that a context-specific response biomarker existed, metabolomic changes from baseline were compared between response categories. A model based on 12 metabolites was constructed that successfully discriminated between the three response categories (Fig 4B). It is possible that this was an underestimate of the performance of the model because of imbalances in the numbers of individuals with each response category. Metabolites that identified individuals with a measurable response are illustrated in Fig 5C.

We examined the biomarkers for commonalities. Only one metabolite (sorbitol) was increased in the two *in vitro* biomarkers as well as the axitinib-specific biomarker. However, in addition, there were metabolites identified *in vitro* that changed in the same way in clinical samples, although they did not appear in the axitinib-specific response biomarker: 5,6-dihydrouracil and glycopyranose increased with response; glutamic acid, glutamine and a lactose derivative decreased with response.

### 2.6. Serial analysis to detect emergence of resistance

If it is possible to detect treatment response based on patterns of change compared to pretreatment baseline, then it may also be possible to detect early evidence of the emergence of chemoresistance (i.e. changes that preempt progression). Samples taken within 2–4 weeks prior to radiographic progression were evaluated, and we examined the behavior of the 12 metabolite meta-biomarker. In general, changes in concentrations of individual metabolites that appeared with response reversed at the pre-progression time point, although only the changes in prolene were statistically significant. However, at the pre-progression time point, the response meta-biomarker is clearly extinguished (Fig 5D). This suggests that disappearance of the response biomarker signifies the loss of benefit of chemotherapy.

### 2.7. Response-related changes in metabolism

There were some common metabolic features that appeared in association with effective doses of antineoplastic agents (Fig 5). Effective treatment generally perturbs energy production, as well as some of the metabolic features characteristic of rapidly dividing cancer cells. Glycolysis and the citric acid cycle are slowed, although surprisingly not in all conditions. Galactose metabolism, a source of glucose, is diminished. There is a reduced consumption of glutamine, a preferred fuel for cancer cells. Reduced utilization of glutamine may be the result of slowed pyrimidine synthesis secondary to slowed proliferation. Amino acid abundance is decreased, and the unavailability of substrate results in the inhibition of the urea cycle.

## Discussion

Treatment-related changes in the metabolome may be secondary to a number of factors, which must all be controlled to derive a biomarker that reflects treatment efficacy. There may be (dose-dependent) pharmacologic effects (on tumor and host); treatment may affect general state of health or organ function; and off-target toxicities may emerge. Contained within that gemisch of treatment-related metabolic changes is a pattern of changes that reflects treatment efficacy. This response biomarker may reflect a diminution of tumor-derived metabolic output (“tumor signal”), reduced cell proliferation, or cell death. Indeed, in our studies, no metabolite or group of metabolites consistently changed with effective treatment, but *patterns* of changes associated with response consistently reflected extinguished hallmarks of cancer cell metabolism and features of diminished cell proliferation.

The idea for following changes in the metabolome to monitor treatment response is not new, but the strategy we employed to detect treatment effectiveness is novel. Other investigators have utilized various analytical platforms to identify chemotherapy-related changes in the metabolome. Tiziani et al. described a nuclear magnetic resonance (NMR)-based method to screen drugs for efficacy by evaluating cellular response to treatment [10]. The method involved quenching metabolic processes, then characterizing the intracellular and extracellular metabolome; this was not applied to clinical samples. A number of investigators have observed treatment-related changes in tumor extracts and blood, but the experimental design did not allow one to clearly identify changes that were related the effectiveness of chemotherapy, as opposed to pharmacologic effects [4–6,11].

A few reports of metabolomic changes that accompany effective chemotherapy have emerged, although in these too the pharmacologic effects on the metabolome have not been subtracted. Treatment-related reductions in the metabolomic “tumor signal” have been described in tumor and blood [12,13]. Higher levels of glutamine in tumor and blood have been associated with tumor inhibition after most, but not all, drugs [5,11,13]. In treated murine melanomas and lung cancers, similar metabolic themes emerged as in our experiments: growth inhibition was associated with accumulation of glucose, glutamine and aspartate (which reflects the downregulation of nucleoside synthesis); and there was a resumption of glutamine utilization in comparison to glucose utilization during the growth recovery phase [11]. Finally, in patients with locally advanced breast cancer, there was an increase in glucose, and a decrease in glycine as well as choline-containing compounds following treatment in tumor biopsies from long term survivors [14].

We have demonstrated the feasibility of detecting a drug-specific response biomarker using a unique discovery approach. The approach consists of procuring serial samples before and during treatment. The “within patient” experimental design (identifying changes rather than monitoring levels of metabolites) is a powerful means of minimizing unexplained inter-individual variability. This and the treatment of metabolites as groups of co-related changes (“metabiomarkers”) reduce noise and enhance specificity.

One intriguing finding is evidence for patterns of metabolomic changes that are stereotypical for particular biological effects. There was a pattern of change characterizing growth inhibition from a variety of agents with diverse mechanisms of action. Another pattern was associated with cell death attributable to miscellaneous agents. Certainly, there are other examples of generalizable metabolic changes. For example, systemic therapy consisting of a wide range of agents can result in diminished 18-fluorodeoxyglucose (FDG) uptake in tumors, reflecting a reduction in glycolysis. Lactate dehydrogenase (which catalyzes the interconversion of lactate and pyruvate) is released from dying cells, which forms the basis of a common cytotoxicity assay. It is too early to speculate whether there is a similar pattern of changes in the metabolome that appears with effective chemotherapy *in vivo*. To derive such a universal response biomarker, large numbers of clinical trial samples collected serially before and during treatment will need to be tested. Drug-specific biomarkers of response should initially be the focus of biomarker discovery. As diverse clinical trials are similarly analyzed, it may be possible to see common and recurrent pattern(s) of change that reliably reflect treatment efficacy.

One challenge with discovering and validating a biomarker for monitoring the efficacy of antineoplastic drugs is how to interpret the stable disease response category. This is particularly problematic for targeted drugs, which tend to be cytostatic, as opposed to cytotoxic. Stable disease could represent a limited response to chemotherapy (ie: stabilization of growth), or it could be a reflection of indolent tumor biology. To discriminate indolent tumor biology and response, larger numbers of patients will have to be analyzed, and metabolomic responses will need to be linked to progression free survival. In our clinical cohort, the presence or absence of the response biomarker in the SD group did not correlate with PFS or OS. The lack of correlation with survivals may have been a reflection of the generally poor prognosis of the patients included in the clinical trial, or it may have been due to insufficient numbers of patients.

Another challenge in the development of a response biomarker is related to timing of blood samples. Ideally, a test would be devised that detects benefit or identifies lack of benefit between the first and second cycle of chemotherapy. That would enable the oncologist to alter the treatment plan before significant toxicities appear. In clinical samples, the changes that most closely correlate with response category appear after the first cycle; at the second cycle, the relationship is less clear (data not shown). Metabolic changes are visible on PET scan after a single cycle of effective chemotherapy [8]. In future work, it will be important to understand the kinetics of any biomarkers that are developed, and these could vary with the drug and tumor type.

One weakness in the approach described is the semi-quantitative nature of the metabolomic data acquired by GC-MS and other mass spectrometry-based platforms. While this is adequate for comparing metabolite levels in various conditions, measurements can be distorted by technical factors. To enhance the accuracy of this approach, internal standards such as deuterium-labeled metabolites will be essential. It is conceivable that the addition of internal standards would reduce measurement variation, which would enhance the accuracy of discriminating classes based on treatment or response.

## Conclusions

We have described treatment-related patterns of changes in the metabolome that signify beneficial antineoplastic effects. The degree of change from baseline reflects the degree of tumor growth inhibition, and response-related changes in metabolites revert back to baseline levels as tumor growth resumes. By analyzing serially collected samples from larger clinical trials, the strategy we have employed can be used to discover and validate biomarkers that reflect effective treatment. More importantly, it will be essential to be able to identify individuals who are not benefiting from a chemotherapeutic agent. This approach could be used as a part of an adaptive treatment algorithm, where chemotherapy modifications are guided by the appearance and disappearance of these biomarkers.

## Materials and methods

### 5.1. Cell lines

Human colorectal cancer (HCT-116 and HT-29), breast cancer (MDA-MB-231 and MCF-7), melanoma (SK-MEL-28), and neuroblastoma (IMR-32) cell lines were purchased from ATCC (Manassas, VA, USA) prior to 2013, and cell stocks were stored in liquid nitrogen. HCT-8 colorectal cancer cells were a kind gift from Dr. Don Fujita (University of Calgary, Calgary, AB) and obtained in 2015. Each cell line was tested for mycoplasma (MD Biosciences Cat# 409010), and mycoplasma negative cell stocks were frozen for experiments. These frozen cells were used for experiments in under 3 passages, and no authentication of cell lines was done.

### 5.2. In vitro experiments

Cells were seeded into 96 well plates in duplicate and allowed to rest for 5 hours before addition of drug. For experiments with antineoplastic agents, each cell line was seeded at a density of 20,000. Antineoplastic agents included: paclitaxel (Hospira, Lake Forest Illinois), irinotecan (Hospira, Lake Forest, USA), oxaliplatin (Sanofi S.A, Paris, France), carboplatin (Hospira, Lake Forest, USA), doxorubicin (Pfizer, New York, USA), 5-fluorouracil (5-FU; Hospira, Lake Forest, USA), sunitinib (Selleckchem, Houston, USA), brivanib (Cedarlane, Burlington, ON, Canada). All antineoplastic drugs except brivanib were kind gifts from Dr. D. Morris (University of Calgary). Cells were exposed to drug for 72 hours, then 50μL of supernatant was collected for metabolomic analysis and growth inhibition was estimated by MTT assay. The supernatant was stored at -80°C until metabolomic analysis.

A “response” to drug (analogous to effective chemotherapy in patients) was considered to have occurred in conditions in which there was a ≥15% growth inhibition (compared to untreated controls). This arbitrary assignment was based on the observation that growth inhibition ≤10% occasionally appeared in cells treated with extremely low drug doses, yet when ≥15% growth inhibition appeared, it reliably marked the beginning of an upward slope on the dose-response curve.

For experiments using various conditions to induce cell death, cells were seeded at a density of 250,000. Agents used to induce cell death included: staurosporine (Sigma-Aldrich, Oakville, ON, Canada), actinomycin D (Sigma-Aldrich, Oakville, ON, Canada), tumor necrosis factor-a (TNF-α; Cedarlane, Burlington, ON, Canada), and dimethyl sulfoxide (DMSO; Sigma-Aldrich, Oakville, ON, Canada) and Triton X-100 (Sigma-Aldrich, Oakville, ON, Canada). In addition, cells were subjected to 15 min of heat in a 70°C incubator at baseline and every 24 hours for 72 hours; or cells were subjected to two freeze-thaw cycles at baseline and every 24 hours, for 72 hours. For each of these conditions, supernatant was collected before addition of drug (or physical agent), then at 72 hours after treatment. Cells were subjected to flow cytometry to estimate proportion undergoing apoptosis or necrosis.

### 5.3. MTT assay

MTT assay was used to reflect the number of viable cells at the end of 72 hours. MTT (3-(4,5-dimethylthiazol-2-yl)-2,5-diphenyltetrazolium bromide) was purchased from Sigma-Aldrich (Oakville, ON, Canada). To each well in a 96 well plate, 200μL of 10% MTT was added and incubated for 3 hours. After addition of DMSO, the absorbance was read on a spectrophotometer at 600 nm. Absorbance of blank wells was subtracted from all measurements. Percent growth inhibition secondary to antineoplastic agents was calculated as the difference between absorbance of untreated cells and absorbance of cells exposed to a range of doses of each drug divided by absorbance of untreated cells x 100.

### 5.4. Flow cytometry

For experiments on agents causing cell death, flow cytometry was used to estimate the proportion of cells that had undergone necrosis and/or apoptosis. At 72 hours, after collection of supernatant, cells were stained with PE-Annexin (BD Biosciences, Mississauga, ON, Canada) and 7-AAD (BD Biosciences, Mississauga, ON, Canada). Flow cytometry was done on a BD Bioscience LSRII Cytometer, and analyzed using the BD Diva software version 6.1.3.

### 5.5. Clinical samples

This study was approved by the Health Research Ethics Board of Alberta (HREBA.CC-16-0239 and HREBA.CC-14-0074). Clinical samples were derived from a phase II clinical trial of second-line axitinib in patients with advanced hepatocellular carcinoma (Clinical trial registration number: NCT01334112) [15]. Sera were collected at baseline and 2–4 weeks after treatment was initiated, then monthly until progression was documented. Response to therapy was estimated by CT scan done at 8 weeks, using Choi criteria [2,15]. In addition, in responding patients, CT scans were repeated every 8 weeks to monitor for progression. Written informed consent was received from all participants to access patient charts, treatment, and treatment response such that it could be reviewed for research purposes.

### 5.6. Gas chromatography-mass spectrometry (GC-MS)

Cell line supernatants and sera from clinical samples were submitted to GC-MS and ran with randomized samples. Methods for sample derivatization and for GC-MS have previously been detailed [16]. GC-MS was performed using an Agilent chromatograph 7890A (Agilent technologies Canada Inc., Mississauga ON, Canada) in conjunction with a Waters time-of-flight mass spectrometer (Waters Corp., Milford, MA, USA). QC samples were run throughout the GC-MS acquisition in set intervals. QC samples were made by combining five random samples in each batch run to monitor instrument response over the experiment. Alkane standards were used throughout the experiment as well to obtain *relative* concentrations of metabolites. Blanks were also used throughout each batch run on GC-MS, which consisted of hexane, the same solvent used to extract metabolites.

### 5.7. Data analysis

We were interested in monitoring changes in relative concentrations of metabolites that occurred in each condition. Relative concentrations of each metabolite were first converted to z-scores to minimize inter-batch variability. Metabolite concentrations from baseline untreated cells were subtracted from every experimental condition. Then, to identify changes in metabolites that discriminated responding cells from non-responding cells, orthogonal partial least squares-discriminate analysis (OPLS-DA) was performed, using *changes* in metabolite concentrations as the dependent variable. In the assessment of OPLS-DA models, R^2^Y represents the goodness of fit of the predictive component (where 1 is a perfect fit), and Q^2^Y represents the predictive variation of the model in a seven times cross validation.

For the *in vitro* experiments leading to biomarkers of response or cell death, a training set consisted of randomly allocated samples comprising 75% of the data set. The remaining 25% of the data set was used as a validation set. Receiver operating characteristic (ROC) curves were generated to visualize the performance of each model.

Clinical samples were analyzed by determining changes of each metabolite over time compared to pretreatment baseline. Initially, a one-way ANOVA was used as a filter to identify metabolites that varied over time (ignoring response class), using a P<0.3 cutoff. Using this more restricted list of metabolites, OPLS-DA was used to compare changes in metabolites that distinguished the three response categories (PR, SD and PD). The final model consisted of metabolites that distinguished between response categories with a VIP≥1. VIP is a measure of the metabolites importance and contribution to the model. Thus to refine and reduce noise caused by metabolites that do not contribute highly to our model, this threshold was used.

## Supporting information

S1 File **Table A In S1 Table**: List of metabolites important in discriminating response from no-response in 5-fluorouracil treated colorectal cancer cells (HCT-116, HT-29 and HCT-8); Table B in S1 File:. List of metabolites important in discriminating response from no-response in oxaliplatin treated colorectal cancer cells (HCT-116, HT-29 and HCT-8); Table C in S1 File: List of metabolites important in discriminating response from no-response in brivanib treated colorectal cancer cells (HCT-116, HT-29 and HCT-8); Table D in S1 File: Response-related metabolite list independent of drug mechanism of action and cell type. This list was generated from experiments with HCT-8, HCT-116, HT-29, MCF-7, MDA-MB-231, SK-MEL-28 and IMR-32 cells treated with an array of chemotherapeutic compounds; Table E in S1 File: List of metabolites important in discriminating death from no-death in MDA-MB-231 and HCT-116 cancer cells treated with a variety of cytotoxic agents. Cytotoxic agents are described in Methods; Table F in S1 File: List of metabolites important in identifying the presence of a response in patients with hepatocellular carcinoma treated with axitinib.
(DOCX)
