# Supplementary material for: A strategy for early detection of response to chemotherapy drugs based on treatment-related changes in the metabolome
Source: PLoS One. 2019 Apr 2;14(4):e0213942. doi: 10.1371/journal.pone.0213942 (PMC6445409; doi:10.1371/journal.pone.0213942)
Supplement: S1 File — Table A In S1 Table: List of metabolites important in discriminating response from no-response in 5-fluorouracil treated colorectal cancer cells (HCT-116, HT-29 and HCT-8); Table B in S1 File:. List of metabolites important in discriminating response from no-response in oxaliplatin treated colorectal cancer cells (HCT-116, HT-29 and HCT-8); Table C in S1 File: List of metabolites important in discriminating response from no-response in brivanib treated colorectal cancer cells (HCT-116, HT-29 and HCT-8); Table D in S1 File: Response-related metabolite list independent of drug mechanism of action and cell type. This list was generated from experiments with HCT-8, HCT-116, HT-29, MCF-7, MDA-MB-231, SK-MEL-28 and IMR-32 cells treated with an array of chemotherapeutic compounds; Table E in S1 File: List of metabolites important in discriminating death from no-death in MDA-MB-231 and HCT-116 cancer cells treated with a variety of cytotoxic agents. Cytotoxic agents are described in Methods; Table F in S1 File: List of metabolites important in identifying the presence of a response in patients with hepatocellular carcinoma treated with axitinib. (DOCX) [file pone.0213942.s001.docx]

Supplementary Tables

Table A in S1 file: List of metabolites important in discriminating response from no-response in 5-fluorouracil treated colorectal cancer cells (HCT-116, HT-29 and HCT-8)

| **Compound** | **Quantification  Ions** | **Retention Index** | **Retention Time** | **Centered and Scaled  Coefficient** |
| --- | --- | --- | --- | --- |
| 5,6-Dihydrouracil | 133 147 160 220 235 | 1140.17 | 7.25022 | 0.0621424 |
| Trehalose | 217 243 271 332 361 | 1945.03 | 15.5572 | 0.0778949 |
| Lactose Derivative | 103 157 204 233 319 | 2010.85 | 16.0811 | 0.0830641 |
| Myo-Inositol (6TMS) | 117 157 189 204 319 | 2073.4 | 16.5688 | -0.0278282 |
| Octadecanoic Acid | 129 203 267 399 487 | 2759.53 | 21.2059 | -0.0438023 |
| Phenylalanine (2TMS) | 91 192 218 266 294 | 1627.06 | 12.6372 | 0.0479321 |
| Asparagine [-H2O] (2TMS) | 100 115 128 201 243 | 1529.08 | 11.6172 | 0.0303722 |
| Lysine (4TMS) | 156 174 230 317 434 | 1936.54 | 15.4583 | 0.0468445 |
| Serine (3TMS) | 100 204 218 278 306 | 1354.53 | 9.78945 | 0.0777447 |
| Threonine (3TMS) | 117 218 219 291 320 | 1379.39 | 10.0719 | 0.0539564 |
| Isoleucine (2TMS) | 102 158 218 232 260 | 1297.74 | 9.10911 | 0.0551153 |
| Ornithine (4TMS) | 142 174 200 258 420 | 1813.19 | 14.3545 | 0.0577393 |
| Glutamine [-H2O] (3TMS) MP | 154 211 227 301 344 | 1598.87 | 12.347 | 0.0278834 |
| Aspartic acid (3TMS) | 202 218 232 306 334 | 1536.34 | 11.7131 | 0.0376232 |
| Tyrosine (3TMS) | 100 179 218 280 354 | 1945.26 | 15.5409 | 0.0365535 |
| Glucopyranose [-H20] (4TMS) | 217 232 257 305 450 | 1773.75 | 13.9745 | 0.0561118 |
| Lactose, alpha- (1MEOX) (8TMS) BP | 169 204 319 361 480 | 2687.95 | 20.8294 | 0.0294972 |
| Cystine (4TMS) | 100 146 218 266 411 | 2292.59 | 18.2082 | 0.0908324 |
| Arginine [-NH3] (3TMS) | 142 157 256 358 373 | 1822.67 | 14.4688 | 0.07669 |
| Uracil, dihydro- (1TMS) | 156 | 1471.53 | 11.1078 | 0.0288995 |
| Erythronic acid (4TMS) | 117 205 220 292 319 | 1553 | 11.8766 | -0.0364032 |
|  |  |  |  |  |
|  |  |  |  |  |
|  |  |  |  |  |
|  |  |  |  |  |
|  |  |  |  |  |
|  |  |  |  |  |
|  |  |  |  |  |
|  |  |  |  |  |
|  |  |  |  |  |
|  |  |  |  |  |
|  |  |  |  |  |
|  |  |  |  |  |
|  |  |  |  |  |
|  |  |  |  |  |
|  |  |  |  |  |
|  |  |  |  |  |
|  |  |  |  |  |
|  |  |  |  |  |
|  |  |  |  |  |
|  |  |  |  |  |
|  |  |  |  |  |
|  |  |  |  |  |
|  |  |  |  |  |

Table B in S1 File: List of metabolites important in discriminating response from no-response in oxaliplatin treated colorectal cancer cells (HCT-116, HT-29 and HCT-8)

| **Compound** | **Quantification  Ions** | **Retention Index** | **Retention Time** | **Centered and Scaled  Coefficient** |
| --- | --- | --- | --- | --- |
| 5,6-Dihydrouracil | 133 147 160 220 235 | 1140.17 | 7.25022 | 0.168066 |
| Trehalose | 217 243 271 332 361 | 1945.03 | 15.5572 | 0.183141 |
| Mannose | 117 129 191 204 217 | 1961.19 | 15.6606 | 0.0949805 |
| Lactose Derivative | 103 157 204 233 319 | 2010.85 | 16.0811 | 0.090427 |
| Octadecanoic Acid | 129 203 267 399 487 | 2759.53 | 21.2059 | -0.0773949 |
| Serine (3TMS) | 100 204 218 278 306 | 1354.53 | 9.78945 | 0.155781 |
| Ornithine (3TMS) (Derivate not found) | 142 204 216 243 348 | 1611.25 | 12.4836 | -0.0638658 |
| Proline (2TMS) | 117 130 142 244 | 1299.7 | 9.15573 | -0.0890216 |
| Aspartic acid (3TMS) | 202 218 232 306 334 | 1536.34 | 11.7131 | 0.0536628 |
| Glucopyranose [-H20] (4TMS) | 217 232 257 305 450 | 1773.75 | 13.9745 | 0.00887685 |
| Erythritol (4TMS) | 205 217 293 307 320 | 1503.73 | 11.4422 | -0.1065 |
| Inositol, myo- (6TMS) | 191 265 305 318 507 | 2086.89 | 16.6722 | -0.0370029 |
| Pyruvic acid (1MEOX) (1TMS) | 89 115 158 174 189 | 1045.34 | 6.04973 | -0.000014808 |
| Pantothenic acid, D- (3TMS) | 157 201 261 291 420 | 1981.37 | 15.8507 | 0.0317547 |
| Fructose | 117 217 230 257 437 | 1822.15 | 14.4114 | 0.0310327 |
| Cystine (4TMS) | 100 146 218 266 411 | 2292.59 | 18.2082 | 0.0187651 |
| Uric acid (4TMS) | 157 229 382 441 456 | 2087.16 | 16.6836 | -0.0436686 |
| Arginine [-NH3] (3TMS) | 142 157 256 358 373 | 1822.67 | 14.4688 | 0.0612037 |

Table C in S1 File: List of metabolites important in discriminating response from no-response in brivanib treated colorectal cancer cells (HCT-116, HT-29 and HCT-8)

| **Compound** | **Quantification  Ions** | **Retention Index** | **Retention Time** | **Centered and Scaled  Coefficient** |
| --- | --- | --- | --- | --- |
| 5,6 Dihydrouracil | 133 147 160 220 235 | 1140.17 | 7.25022 | 0.0616091 |
| Trehalose | 217 243 271 332 361 | 1945.03 | 15.5572 | 0.0377851 |
| Mannobiose | 204 217 243 332 361 | 1959.71 | 15.6785 | 0.0384902 |
| Lactose Derivative | 103 157 204 233 319 | 2010.85 | 16.0811 | -0.0818905 |
| Asparagine (3TMS) | 116 159 188 231 258 | 1669.52 | 13.0273 | -0.0394665 |
| Lysine (3TMS) (Derivate not found) | 84 156 230 258 362 | 1737.53 | 13.6151 | 0.0740047 |
| Serine (3TMS) | 100 204 218 278 306 | 1354.53 | 9.78945 | 0.0527323 |
| Cysteine (3TMS) | 100 116 218 220 294 | 1548.69 | 11.8996 | -0.0542176 |
| Ornithine (3TMS) (Derivate not found) | 142 204 216 243 348 | 1611.25 | 12.4836 | -0.0281 |
| Proline (2TMS) | 117 130 142 244 | 1299.7 | 9.15573 | -0.0793238 |
| Glutamine, DL- (3TMS) | 156 203 245 347 362 | 1767.74 | 13.965 | 0.0320594 |
| Glutamic acid (3TMS) | 128 156 246 348 363 | 1622.08 | 12.5751 | -0.0413825 |
| Glucopyranose [-H20] (4TMS) | 217 232 257 305 450 | 1773.75 | 13.9745 | -0.0363408 |
| Erythritol (4TMS) | 205 217 293 307 320 | 1503.73 | 11.4422 | -0.060088 |
| Sorbitol (6TMS) | 157 217 307 319 331 | 1925.84 | 15.3963 | -0.0418996 |
| Inositol, myo- (6TMS) | 191 265 305 318 507 | 2086.89 | 16.6722 | -0.0506601 |
| Citric acid (4TMS) | 183 211 257 273 375 | 1810 | 14.3571 | -0.0521601 |
| Succinic acid (2TMS) | 129 147 172 247 262 | 1311.42 | 9.29568 | -0.0436771 |
| Phosphoric acid (3TMS) | 211 225 283 299 314 | 1280.5 | 8.89993 | -0.0346602 |
| Allantoin (4TMS) | 188 331 357 431 446 | 1873.37 | 14.954 | -0.0573753 |
| Urea (2TMS) | 87 99 171 189 204 | 1234.9 | 8.43062 | -0.0267685 |
| Aminomalonic acid (3TMS) | 174 218 248 292 320 | 1465.6 | 11.0479 | 0.0458931 |
| Nonadecanoic acid (1TMS) | 117 145 201 355 370 | 2358.91 | 18.6605 | 0.0317394 |
| Fructose | 437 | 1807.21 | 14.3282 | -0.0278333 |
| Cystine (4TMS) | 100 146 218 266 411 | 2292.59 | 18.2082 | 0.0618194 |

Table D in S1 File: Response-related metabolite list independent of drug mechanism of action and cell type. This list was generated from experiments with HCT-8, HCT-116, HT-29, MCF-7, MDA-MB-231, SK-MEL-28 and IMR-32 cells treated with an array of chemotherapeutic compounds.

| **Compound** | **Quantification  Ions** | **Retention Index** | **Retention Time** | **Centered and Scaled  Coefficient** |
| --- | --- | --- | --- | --- |
| Trehalose | 217 243 271 332 361 | 1945.026537 | 15.55716611 | 0.215261 |
| 5,6-Dihydrouracil 2TMS | 133 147 160 220 235 | 1140.166407 | 7.250216423 | 0.157364 |
| Serine (3TMS) | 100 204 218 278 306 | 1354.531139 | 9.789454441 | 0.210685 |
| Proline (2TMS) | 117 130 142 244 | 1299.703054 | 9.155733626 | -0.0530797 |
| Arginine [-NH3] (3TMS) | 142 157 256 358 373 | 1822.670181 | 14.46884457 | 0.0228974 |
| Aspartic acid (3TMS) | 202 218 232 306 334 | 1536.339939 | 11.71311492 | 0.0333545 |
| Glutamic acid (3TMS) | 128 156 246 348 363 | 1622.079465 | 12.57508955 | -0.0250349 |
| Erythritol (4TMS) | 205 217 293 307 320 | 1503.732708 | 11.44216204 | -0.167404 |
| Aminomalonic acid (3TMS) | 174 218 248 292 320 | 1465.600997 | 11.04790293 | 0.105563 |
| L-Glutamine (3 TMS) | 103 117 142 231 315 | 1761.47564 | 13.85560499 | -0.0522438 |
| Cystine (4TMS) | 100 146 218 266 411 | 2292.585831 | 18.20822985 | 0.0535871 |
| Allose | 103 157 189 205 244 | 1841.155283 | 14.23763353 | 0.142352 |
| Lactose Derivative (not exactly identified) | 103 157 204 233 319 | 2010.847588 | 16.08107622 | -0.0163078 |
| Asparagine [-H2O] (2TMS) | 100 115 128 201 243 | 1529.076576 | 11.61719315 | -0.0954265 |
| Lysine (4TMS) | 156 174 230 317 434 | 1936.542971 | 15.45826582 | 0.0557817 |
| Cysteine (3TMS) | 100 116 218 220 294 | 1548.691786 | 11.899632 | 0.0272163 |
| Glycine (3TMS) | 86 100 174 248 276 | 1303.949157 | 9.214592387 | 7.26059E-05 |
| Sorbitol (6TMS) | 157 217 307 319 331 | 1925.839232 | 15.39626455 | 0.0523416 |
| Inositol, myo- (6TMS) | 191 265 305 318 507 | 2086.886208 | 16.67220905 | -0.0175998 |
| Succinic acid (2TMS) | 129 147 172 247 262 | 1311.42396 | 9.295676261 | -0.0288634 |
| Erythronic acid (4TMS) | 117 205 220 292 319 | 1553.000498 | 11.87661431 | -0.127856 |
| Glucopyranose, D- (5TMS) | 117 129 191 204 217 | 1957.931489 | 15.67004773 | 0.140679 |

Table E in S1 File: List of metabolites important in discriminating death from no-death in MDA-MB-231 and HCT-116 cancer cells treated with a variety of cytotoxic agents. Cytotoxic agents are described in Methods.

| **Compound** | **Quantification  Ions** | **Retention Index** | **Retention Time** | **Centered and Scaled  Coefficient** |
| --- | --- | --- | --- | --- |
| 5,6-Dihydrouracil | 133 147 160 220 235 | 1172.6 | 7.42133 | 0.0234917 |
| Purine | 131 207 221 262 350 | 1464.6 | 10.876 | 0.0289764 |
| Trehalose | 217 243 271 332 361 | 1955.22 | 15.5345 | 0.0812304 |
| Maltose | 169 217 243 332 361 | 1965.35 | 15.6257 | 0.0635608 |
| Mannose | 117 129 191 204 217 | 1996.29 | 15.8224 | 0.0126059 |
| Sorbose | 84 174 307 373 547 | 2263.35 | 17.9132 | -0.0122547 |
| Fructose (Derivative) | 156 217 343 415 446 | 2492.49 | 19.4963 | -0.0463443 |
| Tryptophan (3TMS) | 130 202 218 291 303 | 2217.68 | 17.6017 | 0.00265314 |
| Serine (3TMS) | 100 204 218 278 306 | 1374.11 | 9.86527 | 0.0495805 |
| Isoleucine (2TMS) | 102 158 218 232 260 | 1300.06 | 9.05572 | 0.0329158 |
| Methionine (2TMS) | 128 176 202 250 293 | 1531.94 | 11.624 | 0.00890634 |
| Cysteine (3TMS) | 100 116 218 220 294 | 1571.45 | 11.9781 | -0.0673319 |
| Proline (2TMS) | 117 130 142 244 | 1297.74 | 9.06291 | -0.0706821 |
| Valine (2TMS) | 100 144 156 218 246 | 1235.05 | 8.25083 | -0.00659317 |
| Tyrosine (3TMS) | 100 179 218 280 354 | 1941.77 | 15.4243 | 0.0127784 |
| Glutamic acid (3TMS) | 128 156 246 348 363 | 1619.39 | 12.4734 | -0.000952002 |
| Inositol, myo- (6TMS) | 191 265 305 318 507 | 2085.11 | 16.572 | 0.00997541 |
| Succinic acid (2TMS) | 129 147 172 247 262 | 1351.03 | 9.52673 | -0.0558145 |
| Phosphoric acid (3TMS) | 211 225 283 299 314 | 1266.96 | 8.71269 | -0.0740209 |
| Threonic acid (4TMS) | 117 205 220 292 319 | 1593.97 | 12.135 | 0.043969 |
| Lactic acid (2TMS) | 117 133 191 219 234 | 1056.98 | 6.13761 | -0.041982 |
| Hypoxanthine (2TMS) | 193 238 249 265 280 | 1815.35 | 14.3032 | 0.0402272 |
| Urea (2TMS) | 87 99 171 189 204 | 1263.33 | 8.57139 | -0.065512 |
| Aminomalonic acid (3TMS) | 174 218 248 292 320 | 1510.53 | 11.2811 | 0.0430268 |
| Tetradecanoic acid (1TMS) | 117 145 201 285 300 | 1868.49 | 14.7481 | 0.0330218 |
| Heptadecanoic acid (1TMS) | 117 132 145 327 342 | 2158.49 | 17.1312 | 0.0653738 |
| Creatinine (3TMS) | 115 143 171 314 329 | 1580.18 | 12.0483 | -0.0431084 |
| Fructose, D- (5TMS) | 117 204 230 257 437 | 1837.42 | 14.5195 | 0.109377 |
| Sorbose (1MEOX) (5TMS) BP | 217 277 307 335 364 | 1845.37 | 14.6011 | 0.026057 |
| Cystine (4TMS) | 100 146 218 266 411 | 2306.86 | 18.2125 | 0.0146165 |
| Uric acid (4TMS) | 157 229 382 441 456 | 2124.37 | 16.8376 | 0.00671956 |
| Arginine [-NH3] (3TMS) | 142 157 256 358 373 | 1822.14 | 14.3751 | 0.0168358 |

| **Compound** | **Quantification  Ions** | **Retention Index** | **Retention Time** | **Centered and Scaled  Coefficient** |
| --- | --- | --- | --- | --- |
| Sorbitol | 103 157 189 205 244 | 1834.187235 | 13.9833161 | 0.15537 |
| Alanine | 100 116 190 218 233 | 1128.83355 | 6.43270507 | 0.153914 |
| Octadecanoic Acid | 117 129 145 341 356 | 2240.300916 | 17.25161609 | 0.131246 |
| Erythronic Acid | 117 205 220 292 319 | 1533.054506 | 11.1582075 | 0.123585 |
| Quinic Acid | 255 334 345 419 537 | 1847.934642 | 14.10930982 | 0.123387 |
| Proline | 117 130 142 244 | 1312.486927 | 8.681507901 | 0.120142 |
| Cystine | 100 146 218 266 411 | 2285.349929 | 17.56073902 | -0.112102 |
| Benzoic Acid | 77 105 135 179 194 | 1249.410245 | 8.020371951 | -0.115364 |
| Lactose | 103 157 204 233 319 | 2017.315874 | 15.52260481 | -0.129705 |
| Pyruvic Acid | 89 115 158 174 189 | 1048.074554 | 5.559115782 | -0.135837 |
| Isoleucine | 102 158 218 232 260 | 1297.762733 | 8.541285344 | -0.152328 |
| Aspartic Acid | 202 218 232 306 334 | 1513.878349 | 10.97858032 | -0.165108 |

Table F in S1 File: List of metabolites important in identifying the presence of a response in patients with hepatocellular carcinoma treated with axitinib.
